# Internet of Things (IoT) Based Design of a Secure and Lightweight Body Area Network (BAN) Healthcare System

**DOI:** 10.3390/s17122919

**Published:** 2017-12-15

**Authors:** Yong-Yuan Deng, Chin-Ling Chen, Woei-Jiunn Tsaur, Yung-Wen Tang, Jung-Hsuan Chen

**Affiliations:** 1 Department of Computer Science and Information Engineering, Chaoyang University of Technology, Taichung 413, Taiwan; allen.nubi@gmail.com; 2 School of Information Engineering, Changchun Sci-Tech University, Changchun 130600, China; 3 Computer Center, National Taipei University, Taipei 237, Taiwan; wjtsaur@mail.ntpu.edu.tw; 4 School of Physical Therapy, Chun Shan Medical University, Taichung 402, Taiwan; tangyw@csmu.edu.tw; 5 Department of Industrial Education, National Taiwan Normal University, Taipei 10610, Taiwan; jhchen@ntnu.edu.tw

**Keywords:** sensor network, cloud computation, healthcare, body area network, mutual authentication, privacy, untraceability

## Abstract

As sensor networks and cloud computation technologies have rapidly developed over recent years, many services and applications integrating these technologies into daily life have come together as an Internet of Things (IoT). At the same time, aging populations have increased the need for expanded and more efficient elderly care services. Fortunately, elderly people can now wear sensing devices which relay data to a personal wireless device, forming a body area network (BAN). These personal wireless devices collect and integrate patients’ personal physiological data, and then transmit the data to the backend of the network for related diagnostics. However, a great deal of the information transmitted by such systems is sensitive data, and must therefore be subject to stringent security protocols. Protecting this data from unauthorized access is thus an important issue in IoT-related research. In regard to a cloud healthcare environment, scholars have proposed a secure mechanism to protect sensitive patient information. Their schemes provide a general architecture; however, these previous schemes still have some vulnerability, and thus cannot guarantee complete security. This paper proposes a secure and lightweight body-sensor network based on the Internet of Things for cloud healthcare environments, in order to address the vulnerabilities discovered in previous schemes. The proposed authentication mechanism is applied to a medical reader to provide a more comprehensive architecture while also providing mutual authentication, and guaranteeing data integrity, user untraceability, and forward and backward secrecy, in addition to being resistant to replay attack.

## 1. Introduction

### 1.1. Background

Due to the rapid development of network hardware technology, a variety of services and applications that make use of wireless connections such as the LTE, 3G, Wi-Fi, Bluetooth and ZigBee communication technologies have become popular in daily life. One such service is remote medical monitoring and care [1,2]. At the same time, governments have formulated new policies in order to respond to the healthcare requirements of aging populations. Their aim is to build a comprehensive medical network using new wireless technologies such as sensor networks and cloud computation [3]. Their goal is to drive the medical industry, combined with the Internet of Things (IoT), to the next phase of application [4].

In current medical fields, information technology is already used for the secure management of drugs via radio-frequency identification (RFID), patient information and blood information, as well as remote medical monitoring of newborns, and many other applications [5,6,7,8]. However, as populations continue to age, the need for expanded medical care-related applications for elderly people has also grown. Examples of technologies in this field include smart wheelchairs, rural medical care, GPS location, and mobile healthcare, signifying very important development needs. On the other hand, the rapid development of a variety of physiological sensing devices has reduced these in size and improved their energy efficiency, making them suitable for long-term wear by the elderly. These body sensors combined with personal wireless devices form a body area network (BAN) [9,10,11,12]. The personal wireless device collects and integrates personal physiological data, and then transmits the data to the backend of the network for related diagnostics and applications.

This means that when people go to a hospital, the medical staff can obtain relevant medical data from their body sensors using a medical reader. The body sensors will transmit the related sensing data to the personal wireless device, which will transmit the data to the medical reader [13,14,15,16]. The medical staff can then provide these data to a doctor for future reference or immediate medical diagnosis. These data can also be sent to the national medical server to be stored for related statistical big data analysis through cloud technology.

Fortino et al. [17] proposed BodyCloud architecture for body sensor networks (BSN). Their scheme defined a network communication protocol for the communication between the body sensors and the cloud server. Subsequently, Fortino et al. [18] proposed another C-SPINE architecture for body-sensor networks. Their scheme defined a network communication protocol for the communication between different body sensors. They also made the hardware implementation for C-SPINE architecture. Gravina et al. [19] proposed a survey for existing BSN environments, including BodyCloud and C-SPINE architecture.

However, many people still seek to violate the privacy of others, or even harm them. For example, a malicious attacker could send incorrect sensing data to a medical reader, causing an incorrect diagnosis. This could delay treatment, or even result in the death of the patient. In addition, attackers may seek to obtain the sensing data of public figures for blackmail or extortion. Therefore, there must be a complete set of encryption and authentication mechanisms that make it impossible for attackers to obtain and modify such sensitive information in order to protect people’s safety and privacy [20,21,22,23].

Previously, while researchers proposed schemes based on the IoT environment, these schemes were either not for healthcare environments [2,4] or lacked the comprehensive security required for healthcare environments [1,3]. Jr. et al. [2] proposed a session-key establishment scheme between an initiator and a responder for the IoT environment, but they did not mention how the initiator and responder would authenticate each other’s legality. Ray et al. [4] proposed an RFID ownership transfer protocol based on the IoT environment with a comprehensive protocol related to the ownership transfer between two RFID tags; while their protocol achieved mutual authentication between RFID tag and RFID reader, the framework differs from our healthcare environment. The title of Moosavi et al.’s study [1] stated that they were proposing a secure scheme for mobility healthcare based on the IoT environment, but actually they only proposed a challenge-response concept for mobile sensor, smart gateway, and end-user; there is no detailed cryptography description in their article. Yang et al. [3] also proposed a framework for healthcare based on the IoT environment, but their protocol only focuses on the server and the user; they did not make a comprehensive protocol for a body sensor, personal reader, medical reader, and medical server.

He et al. [24] proposed a security mechanism to protect sensitive personal information based on a medical care system such as that alluded to above; their scheme provided a generalized architecture. However, this study found that their proposed scheme still had some vulnerability. First, their proposed security mechanism is not complete; it only considers the protocol between body sensors and personal wireless devices, ignoring the protocol between a personal wireless hub and medical readers. Furthermore, in their proposed scheme, only personal wireless devices authenticate body sensors; since body sensors do not authenticate personal wireless devices, mutual authentication is not achieved.

Based on He et al.’s scheme [24], this study addresses the above vulnerabilities, and adds to this by proposing novel extension architecture, namely an IoT-based design of a secure and lightweight BAN health-care system. The proposed authentication mechanism achieves security, privacy and efficiency.

### 1.2. Security Requirements

The security requirements of a secure and lightweight body area network based on the Internet of Things are listed as follows:

#### 1.2.1. Mutual Authentication

In the information-transmission process, the message receiver must be able to verify the identity legitimacy of the sender. Thus, each party must be able to verify the identity legitimacy of the other party in a BAN authentication environment. If both parties can confirm each other’s identities, then mutual authentication can be achieved [25].

#### 1.2.2. Data Integrity

Any information transferred in an unencrypted network environment is vulnerable to malicious attack in the form of modification, where the message delivered to the receiver is not the original message transmitted by the sender. The integrity of the transmitted data must, therefore, be ensured, and protected against tampering in transit [25].

#### 1.2.3. User Untraceability

Malicious attacks may also attempt to determine a person’s physical location by tracing their personal reader. Thus, a secure BAN authentication environment must prevent such positional tracking [26].

#### 1.2.4. Resisting Replay Attacks

Malicious attacks may also intercept the transmitted message between the personal reader and the medical reader, and then impersonate a legitimate transmitter in order to send the same message to the intended receiver. This constitutes a serious breach of personal data security, and must be prevented by a secure BAN authentication environment [27].

#### 1.2.5. Forward and Backward Secrecy

If the session key between the personal reader and the medical reader is compromised at any point by an attacker, the attacker may use the session key for future malicious communications, or use it to obtain previous messages. A secure BAN authentication environment should thus achieve forward and backward secrecy [27].

The remainder of this paper is arranged as follows. Section 2 gives a brief preliminary introduction and a review of He et al.’s scheme [24]. Section 3 presents the proposed improved secure and lightweight body sensor network based on the Internet of Things for cloud healthcare environments. Section 4 presents a security analysis, efficiency calculation and feature comparison of the proposed scheme and He et al.’s scheme [24], while Section 5 offers conclusions.

## 2. Preliminary Introduction and a Review of He et al.’s Scheme

### 2.1. Preliminary Introduction

#### Elliptic Curve Group

Digital network systems are an indispensable technology in daily life, with massive numbers of documents and information being transmitted over networks every day; thus, measures guaranteeing the security of these messages are very important. Several digital encryption systems have, therefore, been proposed to ensure the security of important documents. In 1985, elliptic curve cryptography [28] was proposed, with a message length shorter than the Rivest–Shamir–Adleman (RSA) encryption system. The following is a brief introduction of the elliptic curve group, and its corresponding mathematical hard problems.

Let $F_{q}$ be a prime finite field, $E/F_{q}$ an elliptic curve defined over $F_{q}$, and P a generator for a cyclic additive group of composite order q. The point on $E/F_{q}$ together with an extra point Θ, called the point at infinity, form a group $G=\{(x,y):x,y\in F_{q};(x,y)\in E/F_{q}\}\cup \{\Theta \}$. G is a cyclic additive group of composite order q. Scalar multiplication over $E/F_{q}$ can be computed as follows: tP=P+P+…+P *t* times.

The following problems exist for the elliptic curve group:

**Computational Diffie–Hellman (CDH) Problem**: Given aP and bP, where a,b∈R, Z×q and P are the generator of G, compute the value abP.
**Decisional Diffie-Hellman (DDH) Problem**: Given aP, bP and cP, where a,b,c∈R, Z×q and P are the generator of G, confirm whether or not cP=abP, which is equal to confirming whether or not c=ab mod q.

### 2.2. Notations

qA k-bit prime$F_{q}$A prime finite field$E/F_{q}$An elliptic curve E over $F_{q}$GA cyclic additive group of composite order qPA generator for the group GsA secret key of the systemPKA public key of the system, PK=sPf(x,y)A polynomial function that f(x,y) equal to f(y,x)$H_{i}()$$i^{th}$ one-way hash functionh()A one-way hash function$r_{i},a,b$*x*’s identity, like a universally unique identifier (UUID) code$ID_{x}$A random number of the elliptic curve group$S_{x}$x’s elliptic curve group signatureTIDA transaction number which changes every round$K_{i}$A polynomial function or elliptic curve-related informationPEKA session key established by personal reader and medical reader$E_{x}(m)$Use a session key x to encrypt the message m$D_{x}(m)$Use a session key x to decrypt the message m$CHK_{x}$x’s verified message$A\overset{?}{=}B$Determines if *A* is equal to *B*dataBody sensor’s related sensing information$c_{i}$The session key and transaction number encrypted sensing data

### 2.3. Review of He et al.’s Scheme

In 2013, He et al. proposed a secure lightweight network admission and transmission protocol for body sensor networks [24]. In their proposed scheme, there are three parties: a body sensor, a personal wireless hub, and a BSN administrator.

#### 2.3.1. Body-Sensor Registration Phase

**Figure 1:**
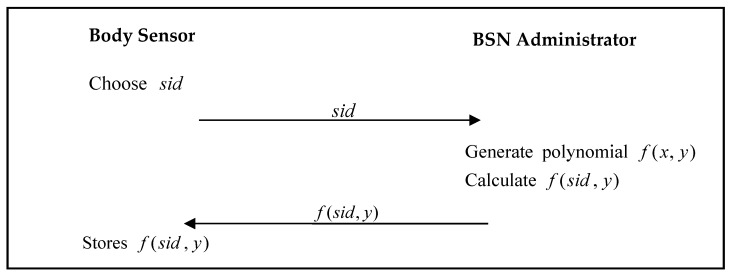
Body sensor registration phase of He et al.’s proposed scheme.

The body sensor must register with the BSN administrator. The body-sensor registration phase of He’s proposed scheme is shown in Figure 1.

Step 1: The body sensor chooses an identity sid, and sends it to the BSN Administrator.
Step 2: The BSN Administrator generates the polynomial f(x,y), calculates f(sid,y), and then sends f(sid,y) to the body sensor.
Step 3: The body sensor stores f(sid,y) in its memory.

#### 2.3.2. Personal Wireless Hub Registration Phase

**Figure 2:**
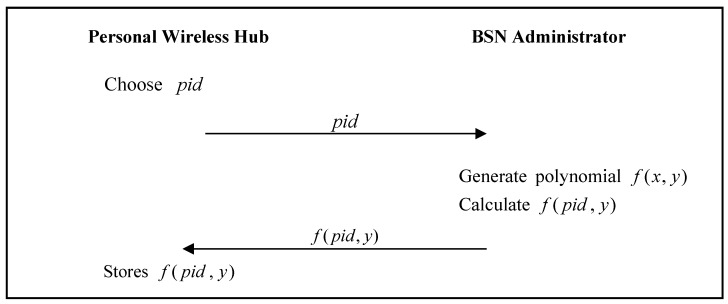
Personal wireless hub registration phase of He et al.’s proposed scheme.

The personal wireless hub must register with the BSN administrator. The personal wireless hub registration phase of He et al.’s proposed scheme [24] is shown in Figure 2.

Step 1: The personal wireless hub chooses an identity pid, and sends it to the BSN Administrator.
Step 2: The BSN Administrator generates the polynomial f(x,y), calculates f(pid,y), and then sends f(pid,y) to the personal wireless hub.
Step 3: The personal wireless hub stores f(pid,y) to its memory.

#### 2.3.3. Authentication Phase

**Figure 3:**
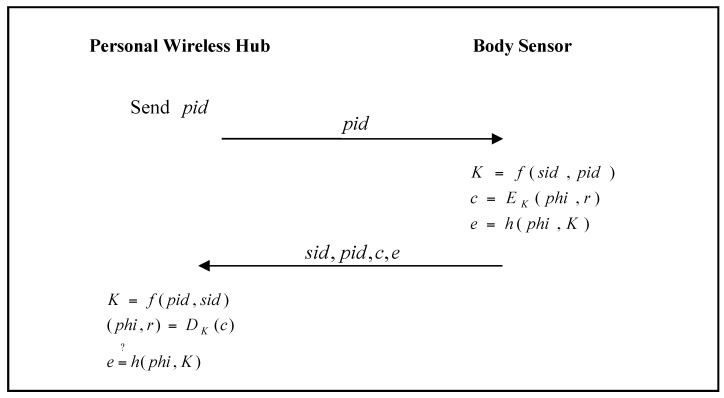
Authentication phase of He et al.’s proposed scheme.

When the personal wireless hub requires related sensor data from a body sensor, it must authenticate the legality of the body sensor. The authentication phase of the proposed scheme is shown in Figure 3.

Step 1: When the personal wireless hub requires related health data from the body sensor, it sends pid to the body sensor.
Step 2: The body sensor calculates K=f(sid,pid), $c=E_{K}(phi,r)$, e=h(phi,K), and then sends (sid,pid,c,e) to the personal wireless hub.
Step 3: The personal wireless hub calculates K=f(sid,pid), $\left(phi,r)=D_{K}(c\right)$, and verifies $e\overset{?}{=}h(phi,K)$ to check the legality of the body sensor. If it passes the verification, the personal wireless hub authenticates the legality of the body sensor, and receives the related health data phi successfully.

### 2.4. Weakness Analysis of He et al.’s Scheme

He et al. proposed a novel scheme for body-sensor network environments [24], but this study found two major shortcomings to their scheme. First, their proposed protocol only focuses on the front end of the BSN environment. They proposed a security protocol between the personal wireless hub and the body sensor. At the back end, the personal wireless hub should also authenticate with the healthcare center in order to protect the health-related data against illegal access. Second, when the personal wireless hub requests related health data, the body sensor sends the encrypted health data directly, without first authenticating the personal wireless hub. In other words, only the personal wireless hub authenticates the legality of the body sensor in their scheme, which means it does not achieve mutual authentication. The attacker may use his/her personal wireless hub to get someone’s health data from that person’s body sensor. A secure BSN environment must achieve mutual authentication between each party.

## 3. The Proposed Scheme

### 3.1. System Architecture

**Figure 4:**
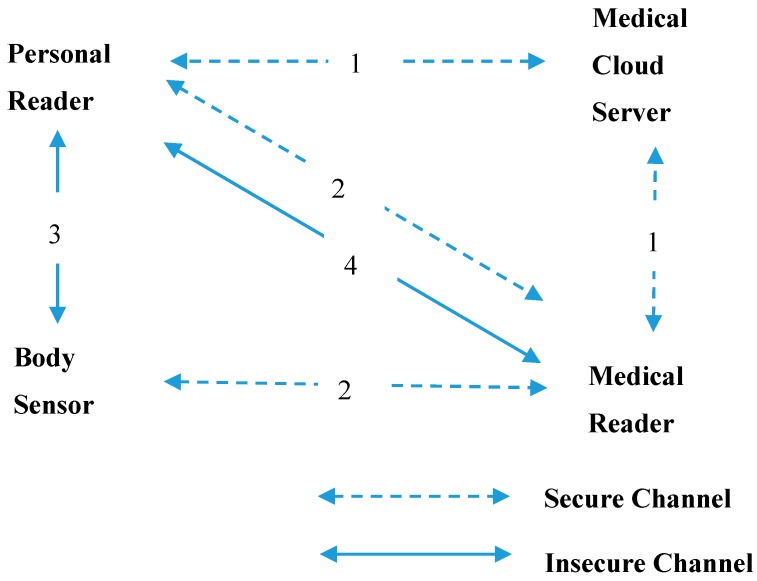
Body area network (BAN) system framework of the proposed scheme.

The BAN system framework of the scheme proposed in this study is shown in Figure 4.

There are four parties in the scheme:

(1) Body sensor: a small sensing device to measure various physiological data of a human body.
(2) Personal reader: a personal reading device carried by an individual; it can receive relevant data from a body-sensor unit, and transmit that data to a medical reader for analysis.
(3) Medical reader: a device carried by medical staff in a medical facility, or by caregivers in a care center; it can receive relevant data from a personal reader for diagnosis by a medical doctor.
(4) Medical cloud server: a cloud server belonging to a national medical institution manages all medical readers and personal readers; all medical readers and personal readers must be registered on the medical cloud server.

1. All personal readers and medical readers must be registered with the medical cloud server through a secure channel. The personal reader and medical reader send their IDs (e.g., universally unique identifier, UUID) to the medical cloud server. The medical cloud server returns information that includes parameters calculated by elliptic curve group technology.
2. All personal readers and body sensors must register with medical readers through a secure channel. The personal reader and body sensor send their IDs (e.g., UUID) to the medical reader. The medical reader returns information that includes parameters calculated by lightweight polynomial function.
3. When a personal reader needs to send related health data to a medical reader, it must first obtain the data from body sensors. After mutual authentication between the personal reader and the body sensor, the personal reader receives the encrypted health data.
4. The personal reader sends its ID and parameters calculated by elliptic curve group technology to the medical reader for authentication. After mutual authentication between the personal reader and the medical reader, the personal reader sends the encrypted health data to the medical reader.

### 3.2. System Initialization Phase

In the system initialization stage, the medical cloud server calculates some parameters, and publishes the public parameters for medical readers and personal readers.

Step 1: The medical cloud server chooses a k-bit prime p, and determines the tuple of elliptic curve group $\left(F_{p},E/F_{p},G,P\right)$.
Step 2: The medical cloud server then chooses s as a secret key, and computes:
  (1) PK=sP
  as a public system key.
Step 3: Finally, the medical cloud server chooses hash function $\left(H_{1}(),H_{2}(),H_{3}(),H_{4}()\right)$, and then publishes $\left(F_{p},E/F_{p},G,P,PK,H_{1}(),H_{2}(),H_{3}(),H_{4}()\right)$ to all medical readers and personal readers.

### 3.3. Body-Sensor Registration Phase

**Figure 5:**
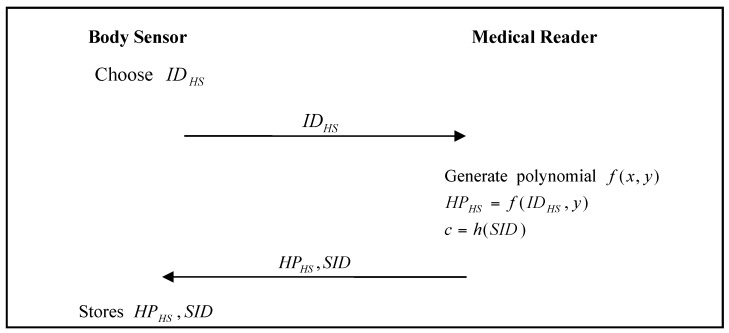
Body-sensor registration phase of the proposed scheme.

The body sensor must register with the medical reader. The body-sensor registration phase of the proposed scheme is shown in Figure 5.

Step 1: The body sensor chooses an identity $ID_{HS}$ (e.g., UUID), and sends it to the medical reader.
Step 2: The medical reader generates the polynomial f(x,y), and calculates:
  (2) $$HP_{HS}=f(ID_{HS},y)$$
  (3) c=h(SID)
  and then sends $\left(HP_{HS},SID\right)$ to the body sensor.
Step 3: The body sensor stores $\left(HP_{HS},SID\right)$ in its memory.

### 3.4. Personal-Reader Registration Phase

**Figure 6:**
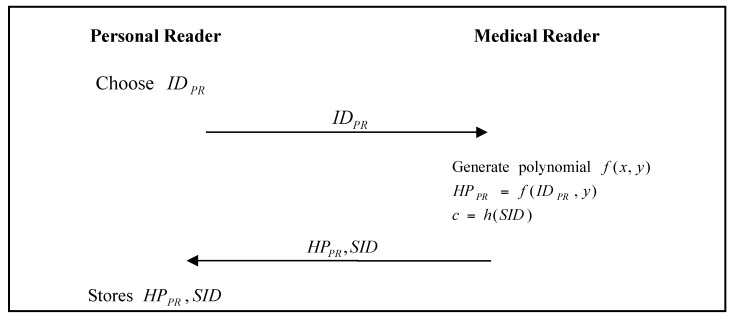
Personal-reader registration phase of the proposed scheme with medical reader.

**Figure 7:**
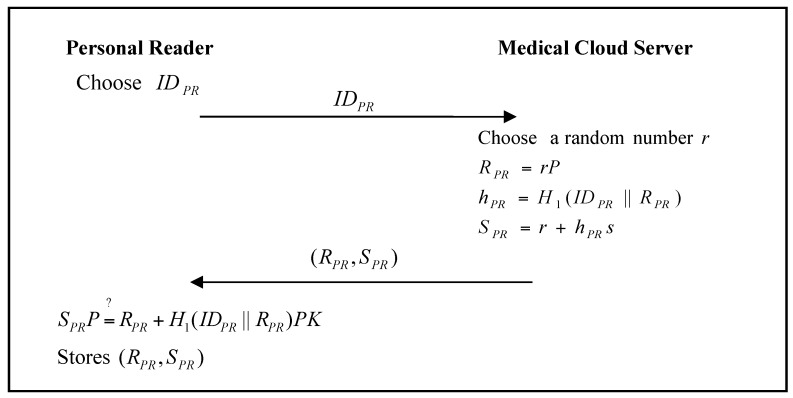
Personal-reader registration phase of the proposed scheme with medical cloud server.

The personal reader must register with the medical reader and the medical cloud server. The personal-reader registration phase of the proposed scheme is shown in Figure 6 and Figure 7.

Step 1: The personal reader chooses an identity $ID_{PR}$ (e.g., UUID), and sends it to the medical reader.
Step 2: The medical reader generates the polynomial f(x,y), and calculates:
  (4) $$HP_{PR}=f(ID_{PR},y)$$
  (5) c=h(SID)
  and then sends $\left(HP_{PR},SID\right)$ to the personal reader.
Step 3: The personal reader stores $\left(HP_{PR},SID\right)$ to its memory.
Step 4: The personal reader chooses an identity $ID_{PR}$ (e.g., UUID), and sends it to the medical cloud server.
Step 5: The medical cloud server chooses a random number r, and calculates:
  (6) $$R_{PR}=rP$$
  (7) $$h_{PR}=H_{1}(ID_{PR}\;\|\;R_{PR})$$
  (8) $$S_{PR}=r+h_{PR}s$$
  and then sends $\left(R_{PR},S_{PR}\right)$ to the personal reader.
Step 6: The personal reader verifies:
  (9) $$S_{PR}P\overset{?}{=}R_{PR}+H_{1}(ID_{PR}\;\|\;R_{PR})PK$$

If it passes the verification, the personal reader stores $\left(R_{PR},S_{PR}\right)$.

### 3.5. Medical-Reader Registration Phase

**Figure 8:**
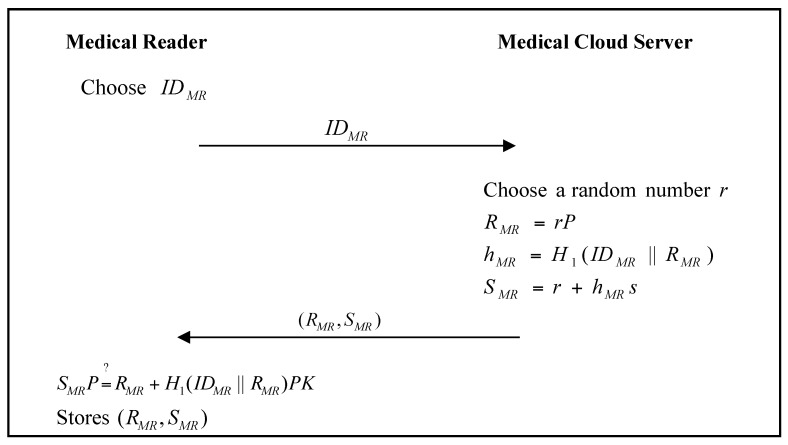
Medical-reader registration phase of the proposed scheme.

The medical reader must register with the medical cloud server. In the proposed scheme, the personal reader and the medical reader can authenticate each other directly without connecting to the medical cloud server. The medical-reader registration phase of the proposed scheme is shown in Figure 8.

Step 1: The medical reader chooses an identity $ID_{MR}$ (e.g., UUID), and sends it to the medical cloud server.
Step 2: The medical cloud server chooses a random number r, and calculates:
  (10) $$R_{MR}=rP$$
  (11) $$h_{MR}=H_{1}(ID_{MR}\;\|\;R_{MR})$$
  (12) $$S_{MR}=r+h_{MR}s$$
  and then sends $\left(R_{MR},S_{MR}\right)$ to the medical reader.
Step 3: The medical reader verifies:
  (13) $$S_{MR}P\overset{?}{=}R_{MR}+H_{1}(ID_{MR}\;\|\;R_{MR})PK$$

If it passes the verification, the medical reader stores $\left(R_{MR},S_{MR}\right)$.

### 3.6. Authentication and Communication Phase

**Figure 9:**
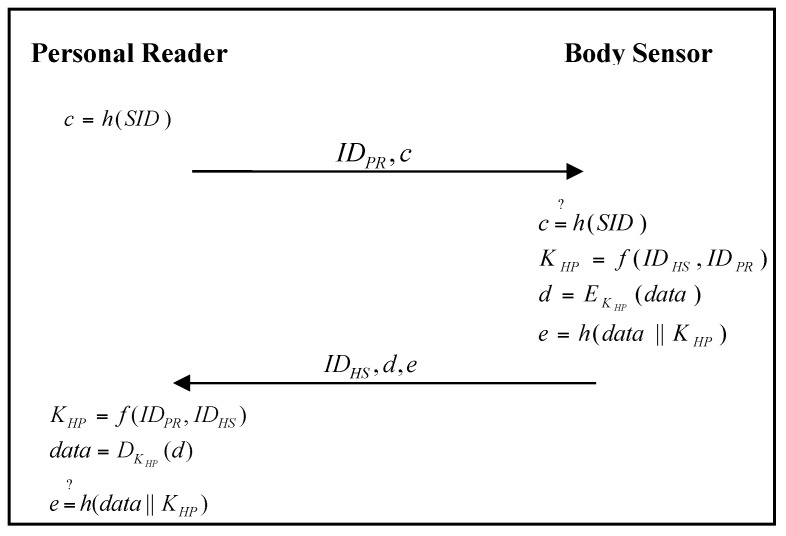
Authentication and communication phase of the proposed scheme for a personal reader and body sensor.

**Figure 10:**
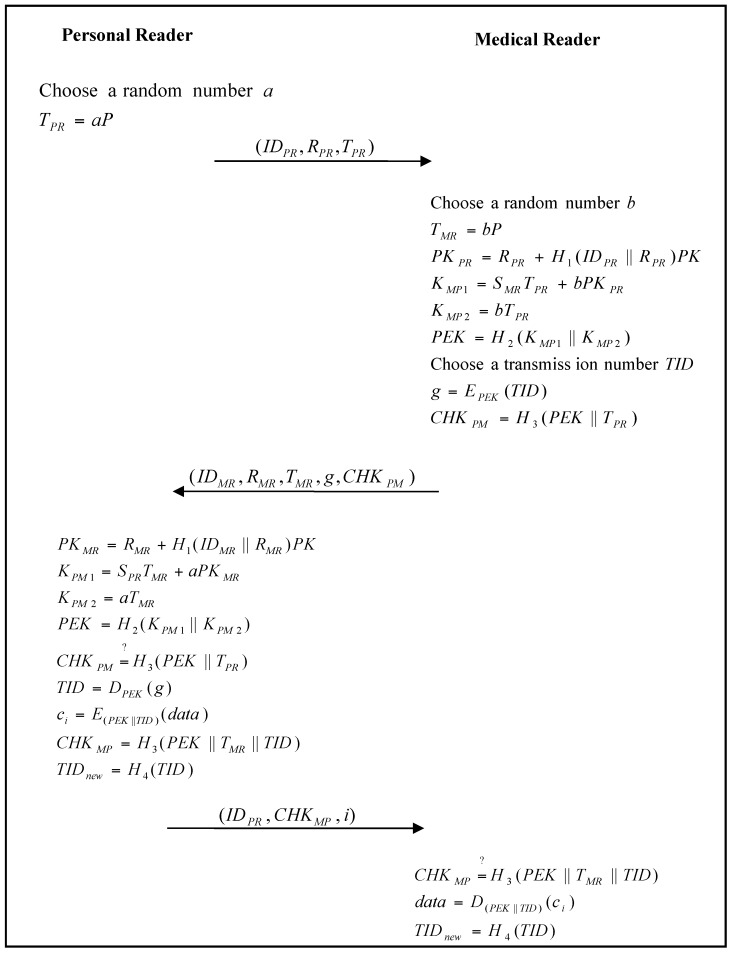
Authentication and communication phase of the proposed scheme for a personal reader and medical reader.

When the personal reader wants to connect to the medical reader for some services, both parties must authenticate each other. In addition, when the personal reader requires related data from the body sensor, they must also authenticate each other. The authentication and communication phase of the proposed scheme is shown in Figure 9 and Figure 10.

Step 1: When the personal reader requires related health data from the body sensor, it calculates:
  (14) c=h(SID)
  and sends $\left(ID_{PR},c\right)$ to the body sensor.
Step 2: The body sensor verifies:
  (15) $$c\overset{?}{=}h(SID)$$
  to check the legality of the personal reader. If it passes the verification, the body sensor calculates:
  (16) $$K_{HP}=f(ID_{HS},ID_{PR})$$
  (17) $$d=E_{K_{HP}}(data)$$
  (18) $$e=h(data\;\|\;K_{HP})$$
  and then sends $\left(ID_{HS},d,e\right)$ to the personal reader.
Step 3: The personal reader calculates:
  (19) $$K_{HP}=f(ID_{PR},ID_{HS})$$
  (20) $$data=D_{K_{HP}}(d)$$
  and verifies:
  (21) $$e\overset{?}{=}h(data\;\|\;K_{HP})$$
  to check the legality of the body sensor. If it passes the verification, the personal reader sends the related health data to the medical reader.
Step 4: The personal reader chooses a random number a, and calculates:
  (22) $$T_{PR}=aP$$
  and then sends $\left(ID_{PR},R_{PR},T_{PR}\right)$ to the medical reader.
Step 5: The medical reader chooses a random number b, and calculates:
  (23) $$T_{MR}=bP$$
  (24) $$PK_{PR}=R_{PR}+H_{1}(ID_{PR}\;\|\;R_{PR})PK$$
  (25) $$K_{MP1}=S_{MR}T_{PR}+bPK_{PR}$$
  (26) $$K_{MP2}=bT_{PR}$$
  and the session key:
  (27) $$PEK=H_{2}(K_{MP1}\;\|\;K_{MP2})$$
Step 6: The medical reader then chooses a transaction number TID, and calculates:
  (28) $$g=E_{PEK}(TID)$$
  (29) $$CHK_{PM}=H_{3}(PEK\;\|\;T_{PR})$$
  and sends $\left(ID_{MR},R_{MR},T_{MR},g,CHK_{PM}\right)$ to the personal reader.
Step 7: The personal reader calculates
  (30) $$PK_{MR}=R_{MR}+H_{1}(ID_{MR}\;\|\;R_{MR})PK$$
  (31) $$K_{PM1}=S_{PR}T_{MR}+aPK_{MR}$$
  (32) $$K_{PM2}=aT_{MR}$$
  and the session key:
  (33) $$PEK=H_{2}(K_{PM1}\;\|\;K_{PM2})$$
  The personal reader verifies:
  (34) $$CHK_{PM}\overset{?}{=}H_{3}(PEK\;\|\;T_{PR})$$
  to check the legality of the medical reader. If it passes the verification, the personal reader calculates:
  (35) $$TID=D_{PSK}(c)$$
  (36) $$c_{i}=E_{\left(PEK||TID\right)}(data)$$
  (37) $$CHK_{MP}=H_{3}(PEK\;\|\;T_{MR}\;\|\;TID)$$
  (38) $$TID_{new}=H_{4}(TID)$$
  and sends $\left(ID_{PR},CHK_{MP},c_{i}\right)$ to the medical reader.
Step 8: The medical reader verifies:
  (39) $$CHK_{MP}\overset{?}{=}H_{3}(PEK\;\|\;T_{MR}\;\|\;TID)$$
  to check the legality of the personal reader. If it passes the verification, the session key PEK between the personal reader and the medical reader is established successfully. The medical reader calculates:
  (40) $$data=D_{\left(PEK||TID\right)}(c_{i})$$
  and also updates the transmission number TID to $TID_{new}$ by:
  (41) $$TID_{new}=H_{4}(TID)$$
  for future communication.

## 4. Security Analysis

### 4.1. Mutual Authentication

In the proposed scheme, when the personal reader wants to communicate with the medical reader, they must authenticate each other. The personal reader uses:

(42) $$CHK_{PM}\overset{?}{=}H_{3}(PEK\;\|\;T_{PR})$$

to verify the legality of the medical reader, and the medical reader uses:

(43) $$CHK_{MP}\overset{?}{=}H_{3}(PEK\;\|\;T_{MR}\;\|\;TID)$$

to verify the legality of the personal reader. Only a legal personal reader or medical reader can calculate the correct session key PEK. The legal medical reader calculates the session key:

(44) $$PEK=H_{2}(K_{MP1}\;\|\;K_{MP2})$$

and the legal personal reader calculates the session key:

(45) $$PEK=H_{2}(K_{PM1}\;\|\;K_{PM2})$$

(46) $$\begin{array}{cc} K_{PM1} & =S_{PR}T_{MR}+aPK_{MR}\\ & =S_{PR}bP+aS_{MR}P\\ & =bS_{PR}P+S_{MR}aP\\ & =bPK_{PR}+S_{MR}T_{PR}=K_{MP1}\\ K_{PM2} & =aT_{MR}=abP=baP=bT_{PR}=K_{MP2} \end{array}$$

Thus, the personal reader can verify the legality of the medical reader, and the medical reader also can verify the legality of the personal reader. The proposed scheme thus guarantees mutual authentication.

Scenario: A malicious attacker uses an illegal medical reader to obtain a patient’s health data from a legal personal reader.
Analysis: The attacker will not succeed because the illegal medical reader has not been registered to the medical cloud server, and it cannot calculate the correct session key PEK. Thus, it will fail when the legal personal reader attempts to authenticate the illegal medical reader. In the proposed scheme, the attacker cannot achieve his/her purpose using an illegal medical reader. In the same scenario, the proposed scheme can also defend against a malicious attack using an illegal personal reader to send fake health data to a legal medical reader because the illegal personal reader has not been registered to the medical cloud server, and it cannot calculate the correct session key PEK. Thus, the attack will fail when the legal medical reader attempts to authenticate the illegal personal reader.

### 4.2. Data Integrity

To ensure the integrity of transaction data, this study uses elliptic curve cryptography to calculate the session key PEK, as well as to ensure data integrity. The malicious attacker cannot use the signatures $\left(K_{PM1},K_{PM2}\right)$ and $\left(K_{MP1},K_{MP2}\right)$ to calculate the correct session key PEK. Only the correct session key will allow successful communication. Thus, attackers cannot modify the transmitted message; therefore, the proposed scheme achieves data integrity.

Scenario: A malicious attacker intercepts the transmitted message from the medical reader to the personal reader, and sends a modified message to the personal reader.
Analysis: The attacker will not succeed because the legal personal reader will use:
  (47) $$CHK_{PM}\overset{?}{=}H_{3}(PEK\;\|\;T_{PR})$$
  to check the data integrity. The attacker cannot calculate the correct session key PEK. Thus, the attack will fail when the legal personal reader authenticates the received message. In the proposed scheme, the attacker cannot achieve his/her purpose by sending a modified message to the personal reader. For the same reason, the attack will fail when the legal medical reader uses:
  (48) $$CHK_{MP}\overset{?}{=}H_{3}(PEK\;\|\;T_{MR}\;\|\;TID)$$
  to check the data integrity. Therefore, attackers cannot achieve their purpose by sending a modified message to the medical reader.

### 4.3. User Untraceability

Another form of privacy attack involves attempting to obtain a person’s physical location by tracing any personal device (in this case, the personal reader). If the personal reader sends the same message continuously, an attacker can trace its location. In the proposed architecture, the response message of the personal reader includes the parameters $CHK_{MP}=H_{3}(PEK\;\|\;T_{MR}\;\|\;TID)$. The random transaction number *TID* is used for every communication round in order to avoid location-tracing. Thus, location privacy is protected, and user untraceability is achieved.

### 4.4. Resisting Replay Attack

Attackers may also intercept the message transmitted between the personal reader and the medical reader. They can attempt to impersonate a legal personal reader or medical reader, and then send the same message again to the intended receiver for a replay attack. Because the transmitted messages are changed every round in the proposed scheme, the same message cannot be sent twice; thus, the replay attack cannot succeed.

### 4.5. Forward and Backward Secrecy

Even if the session key PEK between the personal reader and the medical reader is compromised at any point by an attacker, the system still satisfies forward and backward secrecy. An attacker may use the session key PEK for future communication, or use it to obtain previous messages. However, in the proposed scheme, the session key PEK is randomly chosen by the personal reader and the medical reader, and may only be used in the current round. The attacker cannot use the same session key PEK for future communication, or to obtain previous messages. Thus, a secure BAN authentication environment achieves forward and backward secrecy.

### 4.6. Computation Cost

Table 1 shows the computation costs of the proposed scheme.

From Table 1, the proposed scheme’s computation costs for the medical cloud server, medical reader, personal reader and body sensor in each phase are analyzed. For the highest computation cost in the authentication and communication phase, a medical reader needs five multiplication operations, five hash-function operations, one comparison operation and two symmetric encryption operations. A personal reader needs one polynomial function operation, five multiplication operations, seven hash-function operations, two comparison operations and three symmetric encryption operations. A body sensor needs one polynomial function operation, two hash-function operations, one comparison operation and one symmetric encryption operation. The computation cost and complexity are acceptable.

### 4.7. Communication Performance

The communication cost of the proposed scheme is shown in Table 2.

The communication efficiency of the proposed scheme during the transaction process of each phase was also analyzed. It was assumed that a polynomial function operation required 160 bits, an elliptic curve modular operation required 160 bits, a hash operation required 160 bits, and an advanced encryption standard (AES) operation required 256 bits, while other messages like *id*, *pid*, *random number*, etc., required 80 bits. For example, the authentication and communication phase of the proposed scheme requires four elliptic curve modular messages, four hash messages, three AES messages and five other messages. It thus requires 160 × 4 + 160 × 4 + 256 × 3 + 80 × 5 = 2448 bits. In a 3.5 G environment, the maximum transmission speed is 14 Mbps. This study also considered the authentication and communication phase of the proposed scheme, which only takes 0.175 ms to transfer all messages. In a 4 G environment, the maximum transmission speed is 100 Mbps, and the transmission time is reduced to 0.024 ms (ITU 2016).

### 4.8. Feature Comparison

Table 3 shows the feature comparison of the proposed scheme and He et al.’s scheme [24], and Table 4 is a more detailed mutual authentication comparison of the proposed scheme and He et al.’s scheme.

## 5. Conclusions

Recent developments in sensor-network and cloud computation technology have given rise to what is known as the Internet of Things. Many services can be provided through network cloud environments, including medical care services. In addition, aging populations mean an increased need for expanded healthcare, which has resulted in a new technology development trend. Elderly people can now wear body sensors and personal wireless devices to establish a BAN, which can provide medical care workers and doctors with necessary patient data for diagnoses. However, malicious attackers may seek to obtain sensitive personal data for various reasons. Thus, a robust authentication mechanism for BAN environments that can provide security, privacy and efficiency is necessary.

Previously, He et al. proposed a security mechanism to protect people’s information security in BAN environments [24], but their proposed scheme exhibits vulnerability. This study, therefore, proposes an improved scheme based on their work, which consists of a secure and lightweight body-sensor network based on the Internet of Things for cloud healthcare environments. To sum up, this article mainly achieved the following three contributions: first, we proposed a comprehensive framework for a healthcare BAN environment, including body sensor, personal reader, medical reader, and medical cloud server. Second, we designed secure communication architecture for all roles, unlike the previous work that only mentioned the concept. Third, we showed how the proposed authentication mechanism ensures mutual authentication, data integrity, user untraceability, forward and backward secrecy, and security against replay attacks.

## Figures and Tables

**Table 1:** Computation cost of the proposed scheme.

	Party	Medical Cloud Server	Medical Reader	Personal Reader	Body Sensor
Phase					
Body Sensor Registration Phase		N/A	$1T_{P}+1T_{H}$	N/A	N/A
Personal Reader Registration Phase		$2T_{Mul}+1T_{H}$	$1T_{P}+1T_{H}$	$\begin{array}{l} 2T_{Mul}+1T_{H}\\ +1T_{Cmp} \end{array}$	N/A
Medical Reader Registration Phase		$2T_{Mul}+1T_{H}$	$\begin{array}{l} 2T_{Mul}+1T_{H}\\ +1T_{Cmp} \end{array}$	N/A	N/A
Authentication and Communication Phase		N/A	$\begin{array}{l} 5T_{Mul}+5T_{H}\\ +1T_{Cmp}+2T_{Enc} \end{array}$	$\begin{array}{l} 1T_{P}+5T_{Mul}\\ +7T_{H}+2T_{Cmp}\\ +3T_{Enc} \end{array}$	$\begin{array}{l} 1T_{P}+2T_{H}\\ +1T_{Cmp}+1T_{Enc} \end{array}$

$T_{P}$: Polynomial function operation; $T_{Mul}$: Multiplication operation; $T_{H}$: Hash function operation; $T_{Cmp}$: Comparison operation; $T_{Enc}$: Symmetric encryption operation.

**Table 2:** Communication cost of the proposed scheme.

	Item	Message Length	Round	3.5 G (14 Mbps)	4 G (100 Mbps)
Phase					
Body Sensor Registration Phase		400 bits	2	0.029 ms	0.004 ms
Personal Reader Registration Phase		880 bits	4	0.063 ms	0.009 ms
Medical Reader Registration Phase		480 bits	2	0.034 ms	0.005 ms
Authentication and Communication Phase		2448 bits	5	0.175 ms	0.024 ms

**Table 3:** Feature comparison of the proposed scheme and He et al.’s scheme.

	Scheme	Proposed Scheme	He et al.’s Scheme
Feature			
Mutual Authentication		Yes	No
Data Integrity		Yes	Yes
User Untraceability		Yes	Yes
Resist Replay Attack		Yes	Yes
Forward and Backward Secrecy		Yes	No
Comprehensive Scheme		Yes	No

**Table 4:** Mutual authentication comparison of the proposed scheme and He et al.’s scheme.

	Scheme	Proposed Scheme	He et al.’s Scheme
Feature			
Mutual authentication between body sensor and personal reader		Yes	No
Mutual authentication between personal reader and medical reader		Yes	N/A
